# Regulation of CCL2 Expression by an Upstream TALE Homeodomain Protein-Binding Site That Synergizes with the Site Created by the A-2578G SNP

**DOI:** 10.1371/journal.pone.0022052

**Published:** 2011-07-08

**Authors:** Stephen H. Page, Edward K. Wright, Lucio Gama, Janice E. Clements

**Affiliations:** 1 McKusick-Nathans Institute of Genetic Medicine, Johns Hopkins University School of Medicine, Baltimore, Maryland, United States of America; 2 Department of Molecular and Comparative Pathobiology, Johns Hopkins University School of Medicine, Baltimore, Maryland, United States of America; 3 Department of Neurology, Pathology and Molecular Biology and Genetics, Johns Hopkins University School of Medicine, Baltimore, Maryland, United States of America; 4 The Henry M. Jackson Foundation for the Advancement of Military Medicine, Washington, D. C., United States of America; Fundació Institut Germans Trias i Pujol; Universitat Autònoma de Barcelona CibeRES, Spain

## Abstract

CC Chemokine Ligand 2 (CCL2) is a potent chemoattractant produced by macrophages and activated astrocytes during periods of inflammation within the central nervous system. Increased CCL2 expression is correlated with disease progression and severity, as observed in pulmonary tuberculosis, HCV-related liver disease, and HIV-associated dementia. The CCL2 distal promoter contains an A/G polymorphism at position -2578 and the homozygous -2578 G/G genotype is associated with increased CCL2 production and inflammation. However, the mechanisms that contribute to the phenotypic differences in CCL2 expression are poorly understood. We previously demonstrated that the -2578 G polymorphism creates a TALE homeodomain protein binding site (TALE binding site) for PREP1/PBX2 transcription factors. In this study, we identified the presence of an additional TALE binding site 22 bp upstream of the site created by the -2578 G polymorphism and demonstrated the synergistic effects of the two sites on the activation of the CCL2 promoter. Using chromatin immunoprecipitation (ChIP) assays, we demonstrated increased binding of the TALE proteins PREP1 and PBX2 to the -2578 G allele, and binding of IRF1 to both the A and G alleles. The presence of TALE binding sites that form inverted repeats within the -2578 G allele results in increased transcriptional activation of the CCL2 distal promoter while the presence of only the upstream TALE binding site within the -2578 A allele exerts repression of promoter activity.

## Introduction

Chemokines are small proteins (8–10 kDa) that function as chemoattractants to facilitate the migration of immune cells during immune surveillance and periods of inflammation [1], [2]. CC Chemokine ligand 2 (CCL2; formerly referred to as monocyte chemoattractant protein 1 [MCP-1] recruits monocytes, T-lymphocytes, and basophils to sites of inflammation where further stimulation and production of CCL2 is amplified by a feed-forward cycle [3], [4], [5], [6], [7], [8], [9], [10]. Increased production of CCL2 leads to monocyte-rich infiltrates in organ specific diseases, including the brain, and elevated levels of CCL2 are observed in cerebral spinal fluid in a number of CNS diseases [11], [12], [13], [14], [15], [16]. Overproduction of CCL2 in the myocardium and pulmonary arteries of transgenic mice led to myocarditis and pulmonary vascular inflammation [17]. While overproduction of CCL2 leads to disease progression and severity in inflammatory related pathologies, underproduction leads to immune deficiencies in transgenic mice as demonstrated in CCL2 knockout mice challenged with *S. pneumoniae* [18].

CCL2 is a three-exon gene located at 17q11.2-q12, which is regulated by distal and proximal promoters, each separated by approximately 2.2 kb of DNA [3], [4], [6], [7], [8], [9], [19], [20], [21], [22], [23]. The gene product is a small secretory chemokine expressed by macrophages, lymphocytes, and activated astrocytes, and is actively produced in response to stimuli from cytokines, growth factors, and lipopolysaccharides. The relationship between increased CCL2 expression and disease progression and severity has been linked to a single nucleotide polymorphism (SNP) located at position -2578 in the distal promoter of CCL2 in diseases such as pulmonary tuberculosis, HCV-related liver disease, and HIV-associated dementia [14], [24], [25], [26], [27].

Previously, we reported that following activation of human astrocytes with IL-1β, reporter constructs containing the distal promoter for CCL2 comprising the -2578 G allele had a greater fold induction as compared to the A construct in functional assays [28]. Additionally, we reported that the A-2578G SNP within the CCL2 distal promoter forms a 6-bp site for binding of members of the Three Amino Acid Loop Extension (TALE) protein family, which are transcription factors involved in patterning processes in vertebrates [29]. We also demonstrated that two members of this family, PREP1/PBX2, form complexes and bind this site [28]. PBX2 contains a nuclear localization signal (NLS), and when it dimerizes with PREP1, this signal mediates nuclear localization of PREP1 [30]. In addition, PREP1/PBX2 dimers and PBX2 alone form complexes with the homeobox protein HoxA9 [31]. The region of the distal promoter containing the TALE binding site created by the -2778G SNP is located within an interferon-stimulated response element (ISRE) and has been reported to bind *in-vitro* transcribed and translated interferon regulatory factor (IRF) 1 in gel shift assays [25].

In this study, we modeled proinflammatory conditions by activating astrocytes with IL-1β that results in transcription of the CCL2 gene and secretion of the CCL2 protein as previously described. We demonstrated the binding of PREP1, PBX2, IRF1 and HoxA9 to the CCL2 distal promoter in both the -2578 A and G alleles via chromatin immunoprecipitation (ChIP) assays. Binding of the TALE proteins to the -2578 A allele was unexpected. However, TALE binding sites are often found in multiple copies, orientations, and spacing, or as inverted repeats [32]. Thus, we analyzed the distal promoter sequence of CCL2 and identified an inverted TALE binding site located on the opposite DNA strand 22 bp upstream of the TALE binding site containing the A-2578G polymorphism. We have characterized the binding of the TALE proteins PREP1 and PBX2 as well as HoxA9 and IRF1 to the upstream TALE site in the context of both the -2578 A and G alleles, and we propose a new mechanism for the role of TALE sites and the A-2578G polymorphism in the regulation of CCL2 transcription.

## Results

### Interactions between the CCL2 distal promoter and PREP1, PBX2, HoxA9 and IRF1

**Figure 1:**
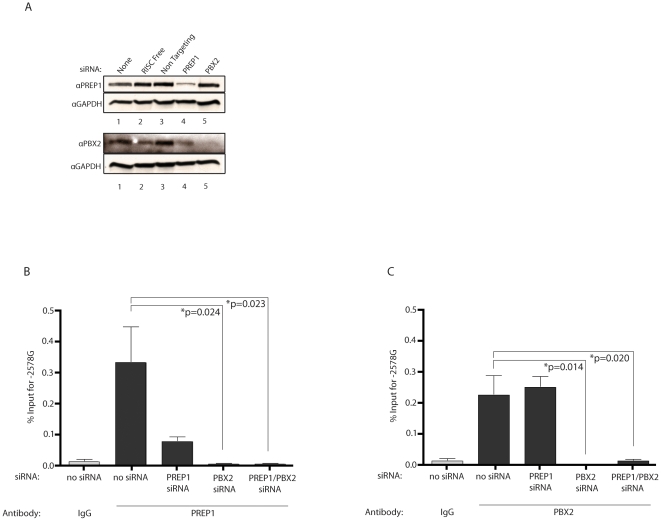
Distinct TALE site binding preferences for PREP1, PBX2, HoxA9 and IRF1 in the CCL2 promoter. ChIP results from U87-MG cells transfected with appropriate plasmids (as described above) and activated with IL-1β for 3 hours. **A.** Western blot results from U87-MG cells transfected with indicated siRNAs. GAPDH represents loading control. **B,C.** ChIP results from U87-MG cells co-transfected with the -2578 G pGL4.11 plasmid and indicated siRNA. Graphics show percent of total DNA immunoprecipitated by each indicated antibody. IgG represents the negative control. Results are expressed as the mean ± S.E. of three independent experiments carried out in duplicate. Differences between multiple groups were tested using ANOVA (Figure 1B, P = 0.0115 and Figure 1C, P = 0.0014). Subsequent pair wise comparisons of groups with each treatment against the control group utilized the Bonferroni correction.

Using ChIP analysis, we measured the interactions between the TALE proteins and the distal CCL2 promoter containing either the -2578 A allele or the -2578 G allele. A human astrocytoma cell line (U87-MG) was activated with IL-1β and transfected with firefly luciferase constructs containing a 929 bp fragment representing the distal CCL2 promoter with either the -2578 A allele or the -2578 G allele. In all experiments, U87-MG cells were activated with IL-1β and produced CCL2 (data not shown). ChIP analysis was followed by quantitative PCR (qPCR; Figure 1A). The binding of transcription factors to the DNA constructs was calculated as a percentage of total DNA precipitated (as described in materials and methods) by a specific antibody and converted to a fraction of the total as described previously (i.e. A/(A+G) or G/(A+G)) [33]. The TALE proteins bound to a greater extent to the -2578 G allele than to the -2578 A allele (Table 1). In addition, we found that there was significantly greater binding of HoxA9 protein and increased binding of IRF1 to the -2578 A allele (Table 1). Increased binding of the TALE proteins to the -2578 G allele was expected since the SNP creates a TALE binding site (Figure 1A); however, it was surprising that the -2578 A allele bound to TALE proteins, PREP1 and PBX2, as well as HoxA9 and IRF1. This prompted us to look at the CCL2 distal promoter more carefully and led us to identify an upstream TALE site present in both alleles.

**Table 1:** Percentage of total CCL2 promoter DNA immunoprecipitated: -2578 A relative to -2578 G.

Precipitating Antibody	-2578 A	-2578 G	Standard Deviation
PREP1	39	61	9.275
PBX2	45	55	21.58
HoxA9	74	26	13.21
IRF1	55	45	9.357

ChIP results from U87-MG cells transfected with -2578 A and G containing plasmids and activated with IL-1β for 3 hours. Results represent the proportional binding of each transcription factor to the -2578 A and -2578 G relative to the total DNA precipitated by each indicated antibody (A/A+G or G/A+G). Anti-IgG antibody was used as a negative control. The standard deviation is the same for both the -2578 A and -2578 G values since their sum is equal to 100. Therefore, the standard deviation is listed only once.

### siRNA-mediated silencing of PREP1 and PBX2 alters TALE site protein occupancy for the -2578 G allele

Since the TALE proteins had a higher percentage of binding to the -2578 G allele, we examined the binding of PREP1 and PBX2 individually and together to the CCL2 distal promoter with the -2578 G allele. U87-MG cells were co-transfected with siRNA and a Firefly Luciferase construct containing a 929 bp fragment with the -2578 G allele. Western blot analyses were used to quantitate the efficiency of PREP1 (protein expression reduced 61% by PREP1-siRNA) and PBX2 (protein expression reduced 97% by PBX2-siRNA) silencing (Figure 1B). ChIP was performed on siRNA-treated samples and data are presented as the percentage of the total DNA that was immunoprecipitated with each antibody for the -2578 G allele. The percentage of immunoprecipitated DNA with anti-PREP antibody was reduced by silencing of PREP1, PBX2 or a combination of PREP1 and PBX2 (Figure 1B). In contrast, silencing of PREP1 protein expression had little effect on the amount of DNA immunoprecipitated with anti-PBX2 antibody. However, the amount of immunoprecipitated DNA was significantly reduced with silencing of PBX2 or a combination of PREP1 and PBX2 (Figure 1C). These data are consistent with previous studies that demonstrate that PREP1 requires dimerization with PBX2 prior to nuclear localization and that the absence of PBX2 prevents nuclear localization and DNA binding of PREP1 [30]. Since PBX2 has been shown to dimerize with other transcription factors, such as HoxA9 [31], it is not surprising that silencing PREP1 does not reduce PBX2 binding to the -2578 G allele. The results confirm that the TALE proteins PREP1 and PBX2 bind to the -2578 G allele and that PREP1 binding is dependent on PBX2.

### Identification of an upstream TALE binding site repeat

**Figure 2:**
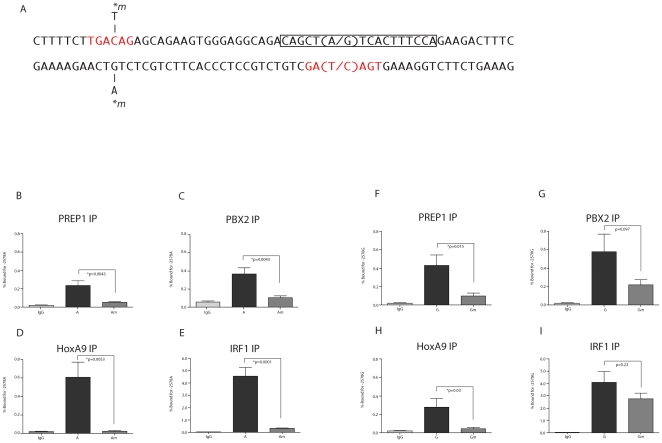
Distinct TALE site binding preferences for PREP1, PBX2, HoxA9 and IRF1 in the CCL2 promoter with and without TALE site repeats. **A.** Sequence of double stranded CCL2 promoter DNA representing the distal regulatory region containing the two TALE binding sites (in red) with the A-2578G polymorphism within parentheses and the incorporated mutation within the upstream TALE site (denoted by *m). The CCL2 ISRE sequence is within the black box. Graphics show the percent of total DNA immunoprecipitated from U87-MG cells by each indicated antibody. **B-I.** U87-MG cells were transfected with either the -2578 A pGL4.11 plasmid or the -2578 A*m* pGL4.11 plasmid. **F-I.** U87-MG cells were transfected with either the -2578 G pGL4.11 plasmid or the -2578 G*m* pGL4.11 plasmid. Percent input was calculated by 100×2∧(Ct_input_ – Ct_enriched_). Input was determined from 1% of the cell lysate and results are expressed as the mean ± S.E. of three independent experiments carried out in duplicate.

The observation that the -2578 A allele bound PREP1 and PBX2 suggested that the CCL2 distal promoter contains either a TALE site or another site that could bind these proteins. Site selection experiments performed by Shen *et al.* demonstrated that the TALE binding sequence TGACAG is usually found in multiple copies with varying spacing, and they frequently form inverted repeats [32]. Based on the results above, we inspected the region proximal to the -2578 polymorphism and identified a second TALE binding site 22 bp upstream of the A-2578G polymorphism (Figure 2A). Site-directed mutagenesis was used to mutate the upstream TALE binding site from TGACAG to TGATAG within the CCL2 promoter constructs containing the -2578 A and G alleles. This mutation is equivalent to the A-2578G polymorphism that had been shown previously to reduce PREP1/PBX2 binding of the TALE binding site [28]. ChIP was performed and the results are presented as the percentages of total DNA precipitated by specific antibodies for either the -2578 A allele or the -2578 G allele (presence of the upstream TALE site mutation designated either A*m* or G*m*) [33]. Mutation of the upstream TALE site reduced binding by both PREP1 and PBX2 for both the -2578 A and G alleles (Figure 2B, C, F and G). While the percentage of DNA bound by either PREP1 or PBX2 decreased proportionately for both the -2578 A and G alleles when the upstream TALE site is mutated, the amount bound to the G allele was consistently higher than bound to the A allele when the upstream TALE site is intact. The percentages of total DNA precipitated by the G allele with PREP1 and PBX2 antibodies were 0.43 and 0.58, respectively, as compared to the A allele (0.23 and 0.37,respectively). These data suggest that the upstream TALE site plays an important role in binding TALE proteins in both the A and G alleles and the presence of the downstream site created by the -2578 SNP increases overall binding of TALE proteins to the distal CCL2 promoter.

Since HOXA9 protein has been shown to form complexes with PBX2 is was not surprising that mutation of the upstream TALE site significantly reduced the amount of CCL2 promoter DNA immunoprecipitated by HoxA9 antibody for both the -2578 A and -2578 G alleles (Figure 2D and H) as was found above for binding of PREP1 and PBX2. However, HOXA9 was found associated with the A allele at greater levels than the -2578 G allele (% immunoprecipitated 0.60 and 0.28, respectively). This suggests that protein complexes associated with the A allele interact with HoxA9 to a greater extent than those of the G allele. When the upstream TALE site is mutated, we found that the extent of the reduction in CCL2 promoter DNA immunoprecipitated by HoxA9 antibody is greater in the context of the A allele (Figure 2D, 25.2 fold) as compared to the G allele (Figure 2H, 6.75 fold). This suggests that the protein associations dependent on the upstream TALE site in the A allele are crucial for HoxA9 interaction.

Finally, immunoprecipitation of the distal CCL2 construct with the mutation in the upstream TALE site with IRF1 antibody significantly reduced the percentage of DNA precipitated for only the -2578 A allele (Figure 2E). While the A and G alleles bind IRF1 equally when the upstream site is intact, mutation of the upstream TALE site leads to a 15-fold (Figure 2E) decrease in binding of the A allele as compared to a 1.5-fold decrease for the G allele (Figure 2I). Since the upstream TALE site is not within an ISRE, this suggests that protein/protein interactions at the upstream site are responsible for the IRF1 binding to the A allele. In contrast, the G allele is within an ISRE site, and binding to the downstream TALE sites appears to contribute equally to the overall IRF1 binding to the distal promtor. Altogether, the data suggest that the upstream TALE site in the A allele contributes to the binding of HOXA9, while in the G allele both TALE sites contribute to binding of IRF1.

### PREP1 and PBX2 differentially regulate transcription of the distal regulatory region containing the A-2578G polymorphism

**Figure 3:**
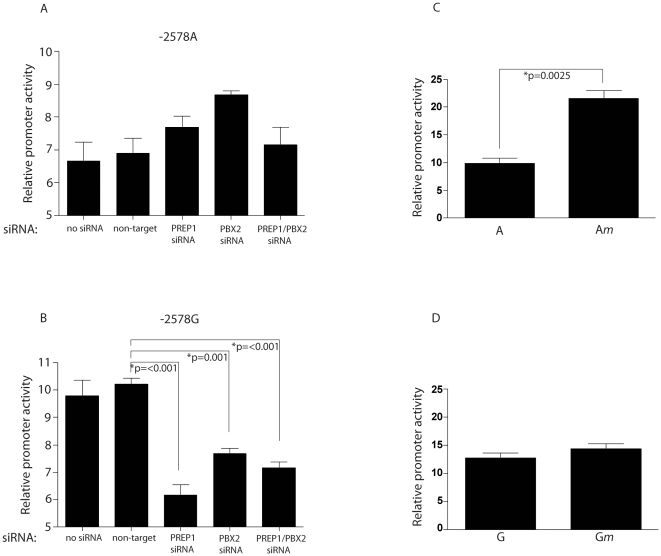
siRNA silencing of PREP1 and PBX2 in transfected U87-MG cells differentially affects promoter activity of the -2578 A and G alleles. **A,B.** Luciferase expression in U87-MG cells transfected with either the -2578 A pGL4.11 plasmid or the -2578 G pGL4.11 plasmid along with indicated siRNAs. **C.** Luciferase expression in U87-MG cells transfected with either the -2578 A pGL4.11 plasmid or the -2578 A*m* pGL4.11 plasmid. **D.** Luciferase expression in U87-MG cells transfected with either the -2578 G pGL4.11 plasmid or the -2578 G*m* pGL4.11 plasmid. All results were normalized to Renilla luciferase values. Results are expressed as the mean ± S.E. of three independent experiments carried out in duplicate. Differences between multiple groups were tested using ANOVA (Figure 3A, P = 0.0495 and Figure 3B, P<.0001). Subsequent pair wise comparisons of groups with each treatment against the control group utilized the Bonferroni correction. Student's two-sided *t* tests were used for 3C and 3D.

To elucidate the effects of the upstream TALE site on transcriptional activity, U87-MG cells were transfected with firefly luciferase constructs containing the distal CCL2 promoter element (929 bp fragment) of either the -2578 A allele or the -2578 G allele. The cells were co-transfected with either PREP1 siRNA, PBX2 siRNA, a combination of PREP1 and PBX2 siRNAs, a non-targeting, control siRNA, or no siRNA. Transcriptinal activity of the -2578 A allele increased following silencing of PREP1 and PBX2 (Figure 3A). In contrast, when both PREP1 and PBX2 were silenced, there was no change in promoter activity. Conversely, silencing PREP1, PBX2, or both PREP1 and PBX2 significantly decreased promoter activity of the -2578 G allele (Figure 3B). These data suggest that the upstream TALE site in the -2578 A allele has a suppressive effect on transcriptional activity when either PREP1 or PBX2 is present. However, when both TALE proteins are limiting, other transcriptional proteins appear to suppress the activity of the -2578 A allele. In contrast, the TALE proteins, PREP1 and PBX2, activate the -2578 G containing the two TALE sites. Taken together these results suggest that the single upstream TALE site suppresses transcriptional activity while the TALE site inverted repeats in the -2578G SNP serve to activate the CCL2 distal promoter.

To further examine the transcriptional effects of the TALE sites in the context of the A and G alleles on the promoter activity, cells were also transfected with firefly luciferase reporter constructs containing the distal CCL2 promoter with a mutated version of the upstream TALE site in the -2578 A or G alleles. The promoter activity of the mutated -2578 A allele (A*m*) was 2.2-fold higher than that of the -2578 A allele, supporting a role in the transcriptional repression of the distal promoter (Figure 3C). However, when the upstream TALE site is mutated in the -2578 G allele there is only a modest increase (p = 0.26) in promoter activity (Figure 3D). These data confirm the observations above that the single TALE site in the context of the -2578 A allele suppresses transcriptional activity of the distal promoter while the upstream TALE site does not have as strong an effect in combination with the downstream TALE site in the -2578 G allele.

## Discussion

In this study we have identified DNA/protein interactions between the CCL2 distal promoter and the TALE family transcription proteins, PREP1 and PBX2 and HoxA9 a binding partner of the TALE proteins as well as IRF1 in both the -2578 A and G alleles. While studies have associated the A-2578G polymorphism in the distal promoter of CCL2 with the progression and severity of numerous inflammatory related diseases [34], [35], [36], the mechanisms responsible for differential regulation of the -2578 A and G alleles are not well understood. Our observation that the TALE proteins bind to the -2578 A allele suggested that an additional TALE binding site might be present in the CCL2 distal promoter and led us to identify another TALE binding site 22 bp upstream of the A-2578G polymorphism. We found that the upstream TALE site in the -2578 A allele suppressed the transcriptional activity of the distal promoter, and suppression was relieved when the site was mutated. In comparison, the presence of the upstream site in the -2578 G allele had little effect on overall transcriptional activity suggesting that the upstream TALE site was not suppressive in the context of the TALE site repeats.

These data suggest that the single TALE site in the -2578 A allele in the CCL2 distal promoter maintains a lower level of transcriptional activity during inflammation while the SNP at -2578 creates an additional TALE site that acts synergistically with the upstream site, relieves the suppression of the single site, and results in a higher level of transcriptional activity of the CCL2 promoter, particularly, during inflammation. We propose that the differential effects of the two sites are likely due to a protein complex including HoxA9 binding only to the upstream TALE site. In the wildtype -2578 A allele, this results in suppression of transcription and lower levels of CCL2 expression. In the -2578 G allele, the SNP creates a downstream TALE site and the suppression is relieved, leading to more transcription and CCL2 expression. This explains the increased levels of CCL2 in individuals who are homozygous for the -2578 G allele.

PREP1/PBX2 complexes have been reported to both activate and repress promoters containing TGACAG motifs in a cell-type dependent manner [37], [38]. This may explain the reason that the -2578 G allele has been reported to have various effects on CCL2 expression in different disease models [24], [25], [39], [40], [41], [42], [43]. These differences may be due to cell-type specific regulation of CCL2 transcriptional regulators and their binding partners. HoxA9 is a unique homeodomain protein with the capability of forming cooperative DNA binding complexes with PBX and PREP1 [31]. In this study, we have demonstrated increased binding of HoxA9 for the -2578 A allele as compared to the G allele. However, binding of both alleles is significantly reduced (25.16-fold reduction for -2578 A and 6.75-fold reduction for -2578 G) following mutation of the upstream TALE binding repeat. This suggests that a complex containing HoxA9 preferentially binds the upstream site and the presence of the downstream TALE site in the -2578 G allele may regulate HoxA9 binding stability to the upstream TALE site. This, in turn, would result in lower overall binding and explain the increased binding to the -2578 A allele.

Previously, IRF1 was reported to bind to an ISRE binding site within the -2578 A allele in gel shift assays [25]. In this report, we corroborated these observations, showing that DNA/protein interactions at both the upstream TALE site and the -2578G SNP TALE site contribute to the binding of IRF1 to the CCL2 distal promoter. We also demonstrated increased binding of IRF1 to the -2578 A allele compared to the -2578 G allele and demonstrated by mutation of the upstream site that both TALE sites contribute to IRF1 binding.

Two NFκB sites are located approximately 50 bp and 80 bp upstream of the TALE site repeats and IRF1 has been shown to interact with NFκB in both gel shift assays and co-immunoprecipitation experiments [44]. If TALE proteins assist in opening the chromatin for NFκB access then the loss of the -2578 site, within the A allele, could limit access to neighboring NFκB sites, which would effectively reduce IRF1 access to the distal promoter and ultimately lead to a loss in promoter activity. Conformational changes in chromatin structure may also modulate TALE site-mediated regulation of the distal and proximal CCL2 promoter. For instance, induction of CCL2 by TNFα leads to conformational changes in the CCL2 promoter facilitating interactions between the two distal NFκB sites and a proximal Sp1 site [6]. A conformational change of this kind may also allow regulatory factors occupying distal TALE sites to interact with proximal factors coordinating CCL2 transcription. However, it is important to note that the -2578G SNP in the CCL2 promoter is the mutation that causes increased expression of CCL2 protein both in vivo in individuals that have the SNP as well as in monocytes and lymphocytes isolated from those individuals compared to those that have either the -2578A homozygous allele or the A/G heterozygous allele [45], [46], [47]. Thus in the context of the entire CCL2 promoter the -2578G SNP shows a dominant role in the context of the NFKB sites.

The degree to which CCL2 is produced is critical as the overproduction of CCL2 may lead to severe inflammation while underproduction can result in suppression of the immune response. In the case of HIV infection, CCL2 facilitates the recruitment of uninfected cells to areas of infection within the brain where infected monocytes may spread the virus [48]. The resulting activation and inflammation contribute to disease severity and progression. Overproduction of CCL2 is also known to increase blood brain barrier permeability, another factor associated with CNS infiltration and inflammation. We have shown that the A-2578G polymorphism creates a second TALE site that relieves suppression of a previously unidentified upstream TALE site and results in increased expression of CCL2. This provides a mechanism explaining the connection between the -2578 G allele, increased CCL2 levels, and disease severity.

## Materials and Methods

### Plasmid Construction

Luciferase expression plasmids containing the distal promoter region were cloned as previously described [28]. Briefly, the 929 bp CCL2 promoter region was amplified by PCR from genomic DNA extracted from U937 cells for the -2578 G pGL4.11 construct (heterozygous A/G) and 293T cells for the -2578 A pGL4.11 construct (homozygous A/A). Sequencing identified appropriate CCL2 distal promoter pGL4.11 clones.

### Site-directed mutagenesis

Mutations involving a TALE-specific nucleotide of the CCL2 distal promoter were generated using QuikChange XL site-directed mutagenesis kit according to the manufacturer's instructions (Stratagene). Briefly, 20 ng of the distal promoter pGL4.11 vectors (described in plasmid production) were used as a template along with the following mutated nucleotide primers: Forward, 5′ –AGGGCATCTTTTCTTGATAGAGCAGA-3′ and Reverse, 5′-CCCACTTCTGCTCTATCAAGAAAAGA-3′. PCR conditions were: predenaturing at 95°C for 1 min, followed by 18 cycles of denaturing at 95°C for 50 s, annealing at 60°C for 50 s, and extension at 68°C for 5 min and concluded with 68°C for 7 min. After digestion with DpnI, 2 µl of PCR product were used to transform XL10-Gold competent cells provided with the kit. Sequencing identified appropriate clones.

### Cell Culture

The human astrocytoma cell line U87-MG was acquired from American Type Culture Collection (ATCC, HTB-14), (Manassas, VA) and were maintained in MEM with glutamine supplemented with 10% FBS, 0.1 mM nonessential amino acids (MEM NEAA), and 1 mM sodium pyruvate. In all experiments, U87-MG cells were activated for 3 hours with recombinant Human IL-1β (201-LB) obtained from R&D Systems (Minneapolis, MN, USA) as described below.

### Transient transfections

DNA Transfections were described previously [28]. Briefly, U87-MG cells in T-75 flasks were transfected with either the -2578 A pGL4.11 plasmid or the -2578 G pGL4.11 plasmid and a pGL4.74 Renilla plasmid (Promega) for normalization control using Lipofectamine 2000 obtained from Invitrogen Corporation (Carlsbad, CA, USA). Transfected cells were incubated for 24 hours and activated with IL-1β for 3 hours prior to harvest. For experiments using siRNA, cells were initially transfected with siRNAs (50 nM; none, Risc-free siGlo, Non-targeting siRNA pool, PKNOX1 (PREP1), and PBX2 pool) using Hyperfect. Transfected cells were incubated for 6 hours at 37°C then media was changed to fresh MDM 10. Transfection was repeated along with plasmid transfection (as desribed above) after incubation for 24 hours at 37°C. Transfected cells were incubated for an additional 24 hours (48 hours total) and activated with 10 ng/mL IL-1β for 3 hours prior to harvest. ON-TARGETplus siCONTROL Non-targeting pool (D-001810-10), siGlo Risc-free (D-001600-01) and ON-TARGETplus Human PBX2 SMARTpool (L-011746-00) siRNAs were obtained from Dharmacon Inc. (Lafayette, CO, USA). PREP1 siRNA (PKNOX1HSS108055).

### Western Blot Analysis

Western blot analysis was performed as described previously [28]. Briefly, whole cell extracts were isolated from U87-MG cells transfected with siRNA. 50 mg of cell lysate was resolved on a 10% Tris-Cl Criterion gel (Bio-Rad Laboratories, Life Science Research, Hercules, CA) and transferred to a nitrocellulose membrane with the criterion wet transfer apparatus. Membranes were washed 5 minutes in TBST, blocked for 20 minutes with 5% milk, and rocked at 4°C overnight with a-PREP1 (1∶2000), a-PBX2 (1∶2000), or a-GAPDH (1∶5000). Secondary antibodies were added at 1∶5000 and blots were developed with SuperSignal Westdura extended duration substrate (Pierce Biotechnology, Rockford, IL).

### Chromatin Immunoprecipitation (ChIP) Assay

ChIP assays were performed as published previously with modifications using the Magna ChIP G Chromatin Immunoprecipitation kits (20–400) obtained from Millipore.Magna ChIP kit (Millipore). PREP1 (sc 6245X), PBX2 (sc 890X), and GAPDH (sc 47724) antibodies were obtained from Santa Cruz Biotechnologies (Santa Cruz, CA, USA). HoxA9 (ab33382-100) antibody was obtained from Abcam Inc., Cambridge, MA, USA. Antibodies for normal mouse IgG (12–371), Briefly, 8×10^6^ transfected (as described above) U87-MG cells were treated with 1% formaldehyde for 10 min at room temperature and neutralized with 1× glycine. Nuclei were isolated from IL-1β activated U87-MGs that had been transfected with either -2578G or -2578A containing vectors with and without the upstream mutation. Samples were sonicated to obtain 500 - 800 bp DNA fragments using the Branson sonicator for 13 12-s pulses at 45% amplitude with 1 min of incubation on ice between pulses. Approxamtely 10^6^ cells were used per immunoprecipitation in a 50 µL volume and pre-cleared with 20 µL Dynabeads protein G (Invitrogen) for 16 hours at 4°C. Pre-cleared cell lysates were incubated with either 4 µg of anti-PREP1, anti-PBX2, anti-HoxA9, anti-IRF1 or mouse IgG (12–371, Millipore) antibody (negative control) at 4°C for 6 hours. Lysates were then incubated with proteinase K at 62°C for 4 hours to reverse cross-linking. Lysates were then incubated at 95°C for 10 minutes to denature proteinase K and DNA was isolated using silica columns. Enriched DNA was quantified using real time quantitative PCR (qRT-PCR) by SYBR-Green (Qiagen) with the following primers to select for construct/insert specific DNA: Forward, 5′-AGGGCATCTTTTCTTAATAGAGCAGA-3, and Reverse, 5-CCTTTGCATATATCAGATAGTAAACAC-3′. Analysis of quantitative qRT-PCR data was analyzed using the percent input method. Percentage of total DNA precipitated was calculated by 100×2∧(Ct_input_ – Ct_enriched_). Input was determined from 1% of the cell lysate and IgG was used as a negative control.

### Luciferase Assays

Luciferase assays using the pGL4.11 Firefly and pGL4.74 Renilla vectors were described previously [28]. The pGL4.11 Firefly and pGL4.74 Renilla vectors and Dual Luciferase Assay System were obtained from Promega Corporation. U87-MG cells were transfected with the appropriate plasmids or plasmids and siRNAs as described above. Cells were then lysed with passive lysis buffer (Dual luciferase assay system; Promega) and transferred to microcentrifuge tubes and sonicated 3× for 20 seconds each at 95% amplitude in a cup horn. Then 20 µL of lysate was subjected to the Dual luciferase assay using the Fluoroskan Ascent FL luminometer.

### Statistics

For all experiments, descriptive statistics (mean and standard deviation) were calculated and compared among groups using parametric methods in PRISM statistical software package. The Student's two-sided t test was used to compare two groups (Figures 2B–I, 3C and 3D). Analysis of Variance (ANOVA) was used to compare multiple groups (i.e., control and three treatments in Figure 1B, 1C, 3A, and 3B). Subsequent pairwise group comparisons were made using Bonferroni's adjustment for multiple comparisons if the overall ANOVA test was determined to statistically significant. Statistical significance was defined as a P value less than 0.05.
